# Process dynamics of serial biases in visual perception and working memory processes

**DOI:** 10.3758/s13423-025-02714-5

**Published:** 2025-05-27

**Authors:** Hyung-Bum Park

**Affiliations:** Institute for Mind and Biology, University of Chicago, 940 East 57 Th St., Chicago, IL 60637 USA

**Keywords:** Serial dependence, Working memory, Mouse trajectory

## Abstract

Serial dependence, a systematic bias in the perceptual decision of current stimuli toward previously encountered ones, has been debated with regard to its locus of processing, with evidence supporting both perceptual processes and post-perceptual working memory (WM) accounts. This study examined the temporal and process-specific dynamics of serial biases across perception and WM processes by analyzing behavioral responses and mouse trajectories. Participants performed two tasks: an immediate perceptual report requiring color matching on a color-wheel with the target color remained visible, and a delayed WM recall consisting of dual responses, a consolidation report immediately after target and mask offset and a retrieval report after a short delay. The results revealed a shift from repulsive biases in immediate perceptual reports to moderate and stronger attraction in WM consolidation and retrieval reports, respectively. Additionally, mouse trajectory analysis further identified a repulsion-to-attraction transition during WM consolidation, suggesting an interplay between sensory adaptation and mnemonic processes. These findings support a mnemonic origin of positive serial dependence, with independent components of perception and WM jointly shaping the output serial bias.

**Supplementary Information:**

The online version contains supplementary material available at 10.3758/s13423-025-02714-5.

## Introduction

Our visual perception remains remarkably stable and coherent despite rapid environmental changes and frequent interruptions from eye movements, blinks, and occlusions. This stability is achieved through serial dependence, a process where the visual system integrates current and past information to create a temporally smoothed representation of the environment. Serial dependence manifests as a systematic bias in the perception of a current stimulus toward previously encountered stimuli (Fischer & Whitney, 2014). This phenomenon extends beyond basic visual features to include a wide range of visual and cognitive attributes, such as facial identity and attractiveness, emotional expressions, and complex object representations (Collins, 2022; Liberman et al., 2014; Manassi et al., 2018; Van der Burg et al., 2019).

While serial dependence may appear detrimental through introducing biases away from the veridical physical attributes, it serves as an adaptive mechanism that exploits statistical regularities of the natural environment to enhance perceptual stability (Cicchini et al., 2018; Kiyonaga et al., 2017). This process resembles proactive interference, where prior information influences the processing of new stimuli. However, unlike traditional proactive interference, which disrupts the distinction between new and old information (Jonides & Nee, 2006; Makovski & Jiang, 2008), serial dependence promotes stability in internal representations across space and time.

The adaptive nature of serial dependence aligns with broader theories in memory research emphasizing its reconstructive nature (Schacter et al., 2011). For example, systematic biases toward prototypical categories in long-term memory enhance mnemonic precision by anchoring uncertain memories to familiar or statistically probable templates (Bae et al., 2015; Won et al., 2023). This reflects the principle that the brain uses prior information not only to maintain stability but also to anticipate and respond to the environment efficiently (Cicchini et al., 2018). By integrating recent sensory history into current percepts, serial dependence reduces noise and transient sensory fluctuations, leading to more stable and coherent perception over time.

Understanding the mechanisms of serial dependence raises important questions about the level of processing at which it occurs. A central debate concerns whether attractive serial dependence originates primarily from perceptual processes or from post-perceptual, particularly working memory (WM), processes (for reviews, see Cicchini et al., 2024; Manassi & Whitney, 2024; Pascucci et al., 2023). The perceptual account posits that serial dependence is an inherent feature of early sensory processing, where the visual system integrates information across successive stimuli to smooth perceptual noise and enhance stability. According to this view, integration occurs almost immediately after stimulus presentation, and supporting evidence includes studies demonstrating attractive biases occurring rapidly during visual processing, even without explicit WM demands (Cicchini et al., 2018; Liberman et al., 2014; Murai & Whitney, 2021; Xia et al., 2016).

In contrast, the post-perceptual account argues that serial dependence emerges when WM processes are engaged (Ceylan et al., 2021; Fritsche et al., 2017; Pascucci et al., 2019). This perspective aligns with the notion that WM bridges sensory experiences over short intervals, maintaining coherent representations despite disruptions (Irwin, 1991). According to this account, serial dependence becomes more pronounced as representational strength degrades, requiring greater reliance on previous information to stabilize current memory contents. Computational models characterize this process as a drift within an attractor network, where current representations are pulled toward recent mnemonic traces (Panichello et al., 2019; Wolff et al., 2020). Consequently, attractive biases are more evident in tasks with longer delays, supporting the idea that serial dependence arises from mnemonic processes rather than immediate perception (Bliss et al., 2017).

This debate often centers on the process-purity of experimental paradigms. For instance, studies supporting the early sensory nature of serial dependence often introduce temporal lags or masking stimuli following target offset, which disrupt the continuous sensory trace and consequently engage WM (Sligte et al., 2010). Conversely, tasks with very short delays between stimulus presentation and response, limiting WM involvement, often report repulsive biases. These repulsion effects, characterized by shifts away from previously seen stimuli, are typically attributed to sensory adaptation (e.g., negative after-effects; Fornaciai & Park, 2019; He & MacLeod, 2001; Moon & Kwon, 2022; Rafiei et al., 2021), further suggesting that attractive serial dependence may be contingent on the engagement of WM.

Neural evidence further supports this distinction. Repulsive patterns observed in early sensory areas during sensory encoding (Hajonides et al., 2023; Sheehan & Serences, 2022; Schwiedrzik et al., 2014; but see John-Saaltink et al., 2016) contrast with the reactivation of previous stimulus predicting behavioral attraction biases in frontoparietal regions (Akrami et al., 2018; Barbosa et al., 2020). Recent neuroimaging work also showed that attraction biases in multivariate neural patterns emerge at later processing stages, indicating that serial dependence involves higher-order cognitive processes (Fischer et al., 2024).

In summary, the repulsion effect observed under certain experimental conditions may serve an important marker for distinguishing different cognitive mechanisms involved in serial biases. According to the post-perceptual WM account, the direction and magnitude of serial biases may vary depending on the extent to which perceptual or WM processes are engaged. Tasks primarily involving immediate perceptual processing are more likely to exhibit repulsive biases due to sensory adaptation, while tasks requiring retention over a short delay engage WM processes and thus result in attractive serial dependence.

The present study aimed to unfold the dynamics of attractive and repulsive serial biases in the continuous estimation of color stimuli,^1^ focusing on the transition from perceptual encoding to post-perceptual WM processes. Specifically, the study focuses on three key aspects: (1) capturing the potential shift from repulsive biases during immediate perception to attractive biases during WM; (2) exploring how serial biases evolve within WM processes, particularly during the distinct phases of consolidation and retrieval after a delay; and (3) investigating the time course of WM consolidation and its effect on the resulting serial biases.

To this end, a novel experimental paradigm that includes both immediate perceptual reports and delayed WM reports was employed. In the perceptual task, participants immediately matched a target color while it remained visible, ensuring continuous sensory input during perceptual analysis and response selection, thus allowing the measurement of perceptual serial bias. In the delayed WM recall task, participants provided two responses within each trial: a consolidation report immediately after target and mask offset, capturing the initial encoding and stabilization of the memory trace, and a retrieval report following a short delay, focusing on memory retrieval over time.

By addressing the process-specific nature of serial dependence, I examined how attractive biases emerge as perceptual information is consolidated into WM. It is well established that WM consolidation is an attention-demanding process requiring time (Park & Zhang, 2024a; Vogel et al., 2006). If the WM account of serial dependence holds, initial repulsive biases in perceptual reports will be overridden by attractive biases in consolidation reports, with the magnitude of attraction increasing over the retention period at retrieval reports.

Moreover, this anticipated repulsion-to-attraction transition should occur gradually during WM consolidation, reflecting the time-resolved integration of perceptual and mnemonic components. To capture these process-specific temporal dynamics arising from opposing sources, I recorded mouse cursor trajectories during each response. Of particular interest were the trajectories during consolidation reports, as their within-response variability provides a fine-grained temporal examination of how repulsive and attractive serial biases rise and fall, depending on the governing cognitive processes at each moment. This approach would support the notion that different cognitive mechanisms, specifically perceptual adaptation and mnemonic integration, jointly shape serial biases in behavioral responses.

## Method

### Participants

Twenty volunteers (11 female) participated in the experiments and received monetary compensation ($20/h). Participants were aged between 18 and 31 years, reported normal or corrected-to-normal visual acuity, and provided informed consent according to procedures approved by the University of Chicago Institutional Review Board.

The sample size was determined based on benchmarks from previous studies on serial biases using similar experimental paradigms (Barbosa & Compte, 2020; Fritsche et al., 2020). Specifically, a priori power analysis was conducted under conventional frequentist criteria using G*Power 3.1 (Faul et al., 2009), aiming to achieve 80% power to detect a medium effect size (Cohen’s *d* > 0.5) with a one-sided test at an alpha level of 0.05 for our primary dependent variable; the amplitude *α* parameter of the first derivative of Gaussian function, determining the direction and magnitude of serial bias. The one-sided test reflected our directional hypothesis that WM tasks would elicit an attraction effect (*α* > 0), to be compared with a repulsion effect (*α* < 0) in perceptual task. It should be noted that this frequentist power analysis was performed to guide sample size planning, but the final data analysis was performed using hierarchical Bayesian modeling. which offers robust estimation of full posterior distributions and credible intervals (CIs) for the parameters of interest (see *Data analysis* for detail).

### Stimuli and procedure

Stimuli were generated in MATLAB (The MathWorks, Natick, MA, USA) using the Psychophysics toolbox (Brainard, 1997). The stimuli were presented on an LCD computer screen (BenQ XL2430 T; 120-Hz refresh rate; 61-cm diameter screen; 1,920 × 1,080 pixels) with a gray background (15.1 cd/m^2^) and positioned approximately 70 cm from the participants.

**Fig. 1:**
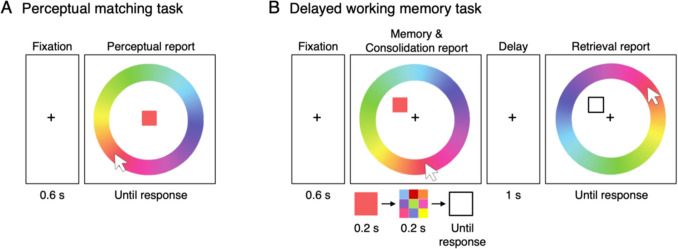
Experimental paradigms for the perceptual matching task and the delayed working memory task. (**A**) In the perceptual matching task, participants viewed a central colored square and matched its color by clicking on a surrounding color-wheel using a mouse. The target remained visible until response. The color-wheel was randomly rotated each trial to prevent strategic mapping of color to spatial location. (**B**) In the delayed working memory task, a single colored square appeared at a random peripheral location as a memory item, followed by a pattern mask. Immediately following the mask offset, participants made a consolidation report by selecting the remembered color on a color-wheel. After a short delay, they made a retrieval report, selecting the same color again on a newly rotated color-wheel. The mouse cursor at each response phase was reset to the central fixation point. Mouse trajectories during responses were recorded from movement onset to click

The experiment consisted of two main tasks: a perceptual matching task and a delayed WM task (Fig. 1). In the perceptual matching task, each trial began with a 600-ms central fixation, followed by a perceptual matching array. The array consisted of a central target colored square (2.0° × 2.0° visual angle) and a continuous color-wheel (radius of 8.2°, thickness of 2.2°) displaying 180 colors evenly distributed in the CIE LAB color space (Zhang & Luck, 2008). All colors had comparable luminance and varied mainly in hue and slightly in saturation. Participants were instructed to report the color of the target square by clicking on the color-wheel using a mouse. Importantly, the target remained visible until the participant responded. The response color-wheel was randomly rotated on each trial. Critically, the target color for each trial was systematically varied in steps of 36° relative to the target in the previous trial, resulting in nine possible relative color difference conditions (i.e., –144°:36°:144°). Additionally, a random color difference condition was included where the target color was randomly sampled from the circular color space. This random condition appeared approximately every ten trials to shuffle the relative target color differences across trials in order to prevent repetition of the same nine color hues for each participant. Data from the random condition were excluded from analysis. Participants were unaware of the systematic manipulation of color differences between trials.

In the delayed WM task, each trial also began with a 600-ms central fixation, followed by a memory array containing a single colored square and a color-wheel. The memory item was presented at a randomly selected location from six possible positions on an imaginary circle with a 4.3° radius from the center. The memory item was displayed for 200 ms, followed by a 200-ms masking stimulus (2.2° × 2.2°) of nine colored squares arranged in a 3 × 3 grid, with colors randomly sampled from the circular color space without repetition, excluding the memory color. Immediately after the mask offset, the memory item was replaced by a black-outlined square, and participants were asked to report the memory color on the color-wheel (i.e., consolidation report). Once they made the consolidation report, a 1-s blank interval followed. After this delay, another test array appeared, consisting of the outlined square at the memory item location and a response color-wheel. Participants were then asked to report the same memory color again (i.e., retrieval report). Notably, the color-wheel was randomly rotated relative to the earlier consolidation report. This random rotation ensured that the spatial mapping of colors on the wheel was different for the retrieval report, thereby minimizing any direct carryover of the consolidation response. The placeholder remained as an outlined square until the end of trial.

For each mouse-click response on the color-wheel in both tasks, the cursor position was reset to the center of the screen, and the cursor trajectories were recorded throughout the response. Each participant completed 150 trials of the perceptual matching task and 400 trials of the delayed WM task. The delayed WM task had 20-s short breaks after every 30 trials.

### Data analysis

#### Hierarchical Bayesian modeling of serial biases

A response error on each trial was measured as the angular distance between the color chosen by participants and the target color. To assess serial bias, the circular mean errors were computed as a function of the relative target color conditions. The target color of the current trial was set to 0°, with negative values indicating counterclockwise (CCW) and positive values indicating clockwise (CW) color that the previous trial’s target had. This approach allowed us to visualize and quantify either repulsive or attractive serial biases. Specifically, negative/positive mean circular errors for those CCW/CW color difference conditions indicate attractive serial bias, respectively, whereas positive/negative mean circular errors for the CCW/CW color difference conditions indicate repulsive serial bias. For the continuity of circular feature space and robust goodness of model fit, the circular mean errors from the two edges, –144° and 144°, were averaged to synthesize the ± 180° condition, resulting in a total of ten color difference conditions.

To further quantify the direction and magnitude of serial biases, I fitted the circular mean errors as a function of the relative color differences with the first derivative of Gaussian (DoG) function, given by

$$y=\alpha \bullet wx\bullet c{e}^{{-\left(wx\right)}^{2}}+\beta$$

where *y* is the circular mean error, *x* is the relative color of the previous trial, and *α* is the amplitude of the peak of the curve. Of the primary interest, the amplitude *α* determines the direction and magnitude of serial bias, specifically, a positive *α* indicates attraction bias and a negative *α* indicates repulsion bias. The parameter *w* scales the width of the Gaussian derivative, the constant $c=\sqrt{2}/{e}^{-0.5}$ is a normalization constant, and the intercept *β* accounts for any overall bias regardless of conditions.

The DoG modeling and parameter estimations were performed using a hierarchical Bayesian model (HBM). This approach could be particularly advantageous in the context of serial dependence research, where the data often involve limited observations per experimental condition due to the necessity of covering a wide range of relative color differences (e.g., a few tens of trials for each of nine conditions in the current experiment). The hierarchical nature of the model allows estimation of population-level parameters while simultaneously accounting for multiple levels of variability in the data structure, such as participant-level as random effects and condition-level as fixed effects (i.e., the types of reports), leading to more stable and reliable parameter estimates. For example, the population-level parameter for the amplitude *α*_*ij*_ is modeled as a function of both participant and condition effects: *α*_*ij*_ ~ *Normal*(*α*_*i*_ + *α*_*j*_, *δ*^2^), where *δ*^2^ describes the variability of participant-by-condition interactions.

I used Markov chain Monte Carlo (MCMC) sampling to estimate the posterior distributions of the parameters, generating 12,000 samples after 12,000 warm-up iterations. This This yielded a matrix of population-level parameter posteriors sized 12,000 × 20 × 3 values (i.e., samples × participants × report types). Model convergence assessed by *R̂* was found to be close to or equal to 1.0 for all population-level DoG parameters (Gelman & Rubin, 1992). Furthermore, the HBM approach was applied to the DoG modeling for the mouse trajectory data, see *Mouse trajectory analysis* section below for detail, including the time evolution (e.g., every 5% time points from movement onset to final response). For this four-level HBM, I took 2,000 samples after 2,000 warm-ups, resulting in a matrix of population-level parameter posteriors sized 2,000 × 20 × 3 × 20 values (i.e., samples × participants × report types × time points).

I chose noninformative and reasonably informative priors to guide the estimation process. Statistical inference was based on posterior means as point estimates and 95% credible intervals (highest density interval, HDI_95%_) which are similar to frequentist 95% confidence intervals. The range of HDIs indicates the strength of evidence depending on whether the upper or lower bound of HDI_95%_ for the experimental effect crosses zero (Kruschke, 2015).

### Mouse trajectory analysis

The mouse trajectories during each of the perceptual, consolidation, and retrieval reports were normalized in time,^2^ with movement onset defined as 0% and the final clicking response set to 100%. Movement onset was determined by a cursor deviation of more than ten pixels in any direction from the starting point at the center. The target color on the continuous color-wheel was rotated to align perpendicularly with the x-axis, allowing any trajectory deviation from the straight line to the target to be represented in the horizontal dimension.

Summary statistics of trajectory deviation were calculated using the area under the curve (AUC), measured in pixels squared per unit time (px^2^/t). This value represents the definite integral of the horizontal cursor offsets over the entire response movement, thereby providing a stable measure of overall trajectory curvature. The pattern of mean AUCs as a function of the relative target color provides a visualization similar to circular mean errors, where the direction and magnitude of serial biases could be quantified by fitting to the DoG function.

Furthermore, two versions of AUCs were calculated based on whether the endpoint bias was included or excluded. Including endpoint bias in the AUC calculation captures the overall bias present at the end of the movement, reflecting the cumulative effect of biases throughout the trajectory. However, midway trajectory curvature does not necessarily exhibit the same direction as the endpoint bias at the clicking response (Park & Zhang, 2022). Instead, in the context of serial biases, the temporal evolution of bias during response, whether repulsive or attractive, appears sensitive to the dynamics of the underlying target representation. Excluding endpoint bias focuses solely on the trajectory path without incorporating the final cursor position. By comparing the AUCs derived from both methods, any shift in bias direction between the response trajectory and the final clicking response, particularly in WM consolidation reports, can be identified.

Additionally, the analysis was extended to capture the moment-by-moment evolution of serial biases. Specifically, the pattern of horizontal cursor deviations at each time point during which the cursor movement progresses as a function of the relative target color difference were again fitted to the hierarchical Bayesian DoG function, with an additional layer of 20 time points in the model structure (i.e., every 5% from the movement onset to final response). This approach generated continuous probability distributions of the DoG function along the response trajectories, thereby allowing for a detailed examination of how different types of serial biases manifest and evolve across time and various cognitive processes.

## Results

### Behavioral reports

**Fig. 2:**
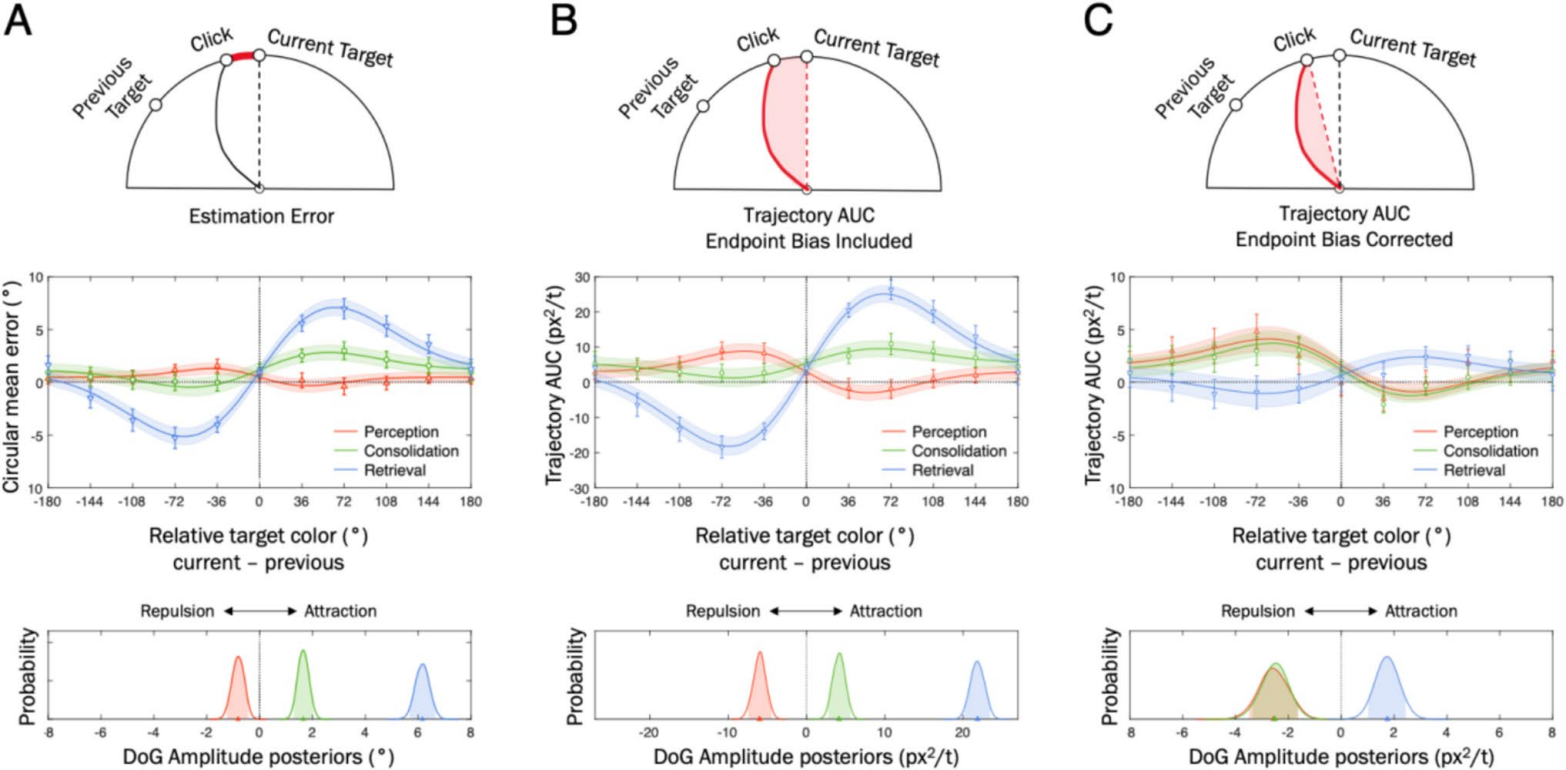
Hierarchical Bayesian Derivative of Gaussian (DoG) modeling of serial biases, measured by circular mean error (***A***), trajectory area under the curve (AUC) with endpoint bias (***B***) and without endpoint bias (***C***), as a function of relative target color differences for perceptual, consolidation, and retrieval reports. Negative values on the x-axis indicate that the previous target color was counterclockwise relative to the current target, while positive values indicate it was clockwise. Error bars represent the standard error of the mean (SEM). DoG fitted curves are averaged across individual-level posteriors of the DoG parameters. The bottom panels summarize the population-level posteriors of the amplitude parameter for each report type. Negative and positive values indicate repulsive and attractive serial biases, respectively. The shaded area under the curve represent 95% highest density interval of the posteriors

The summary of circular mean errors for perceptual, consolidation, and retrieval reports, plotted against the relative target color (i.e., current – previous trial) is shown in Fig. 2A, along with the DoG fit curves. The hierarchical Bayesian analysis of the DoG amplitude (*α*) parameter revealed distinct patterns of serial biases across different phases. For perceptual reports, the posteriors of *α* parameter were credibly negative with its HDI_95%_ not crossing over zero, –0.81° [HDI_95%_ hereafter: –1.24°, –0.43°], indicating a repulsive bias away from the previous target color. In contrast, WM consolidation reports showed a credibly positive serial dependence, + 1.65° [+ 1.29°, + 2.02°], with the magnitude of this attraction bias increased further in retrieval reports, reaching + 6.16° [+ 5.67°, + 6.65°]. The non-overlapping credible intervals across the three reports provide strong evidence that the bias significantly changes over time. These suggest a gradual evolution of serial biases, shifting from repulsion during perception to attraction during WM processes, consistent with previous literature (Bliss et al., 2017).

**Fig. 3:**
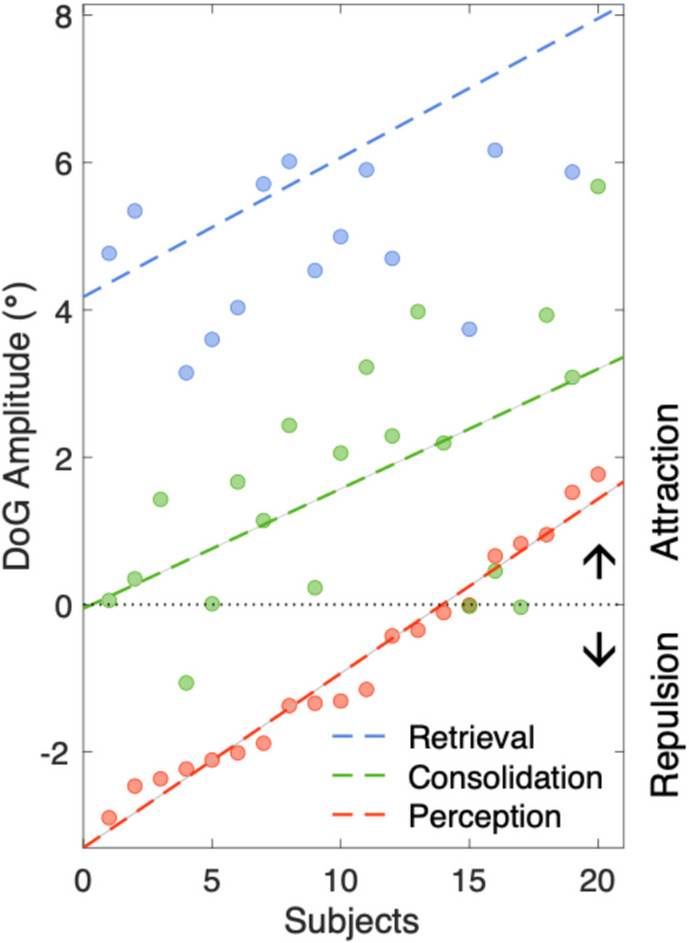
Individual differences in the estimated serial biases. Posterior mean Derivative of Gaussian amplitude parameter values for each participant are plotted separately for perceptual (red), consolidation (green), and retrieval reports (blue), with the linear fitted lines. Individuals are rank-ordered on the x-axis based on their mean amplitude values for perceptual reports, from most negative (strongest repulsive bias) to most positive (attractive bias), illustrating positive relations of serial biases across all three types of reports

The pattern of bias evolution was consistent across participants, though with notable individual differences in the overall magnitude of serial biases. Figure 3 illustrates individual participants’ mean *α* parameter values for each report type, rank-ordered by their mean *α* for perceptual reports. While the majority of participants (15 out of 20) exhibited predominantly repulsive biases in perceptual reports, the remaining participants showed small attraction biases. Interestingly, the individual *α* values across all three types of reports were positively correlated, *rs*(18) > 0.55 [CI_95%_: 0.14, 0.80], *p*s < 0.012. These findings suggest potentially independent-yet-additive mechanisms driving the transition from repulsion to attraction, supporting the hypothesis that serial biases are modulated by both perceptual and mnemonic components. This indicates that while sensory adaptation may initially drive repulsive biases, mnemonic integration during the WM consolidation and maintenance period enhances attractive biases, contributing to the overall evolution of serial dependence.

#### Serial biases reflected in mouse trajectories

The same hierarchical Bayesian DoG modeling was applied to trajectory deviations, separately analyzing two different AUC estimates by including and excluding endpoint bias (see *Data analysis* for details). The DoG-fitted AUCs largely mirrored the behavioral findings (Fig. 2B). Specifically, perceptual reports showed a repulsive bias, –5.98 px^2^/t [–7.49, –4.44], while consolidation reports exhibited moderate attractive biases, + 4.15 px^2^/t [+ 2.77, + 5.63], with retrieval reports showing the strongest attraction, + 21.84 px^2^/t [+ 20.19, + 23.48]. Interestingly, when controlling for bias in the final response by correcting for endpoint bias (Fig. 2C), opposite bias directions were revealed exclusively in consolidation reports. Specifically, while the endpoint bias for consolidation reports showed an attraction bias, the trajectory itself exhibited a repulsion bias, –2.51 px^2^/t [–3.38, –1.63], similar in magnitude to that observed in perceptual reports, –2.55 px^2^/t [–3.51, –1.62]. Retrieval reports again showed attraction effect, + 1.74 px^2^/t [+ 1.00, + 2.45]. This suggests the role of mnemonic processes in shaping attractive serial dependence as WM consolidation progresses.

**Fig. 4:**
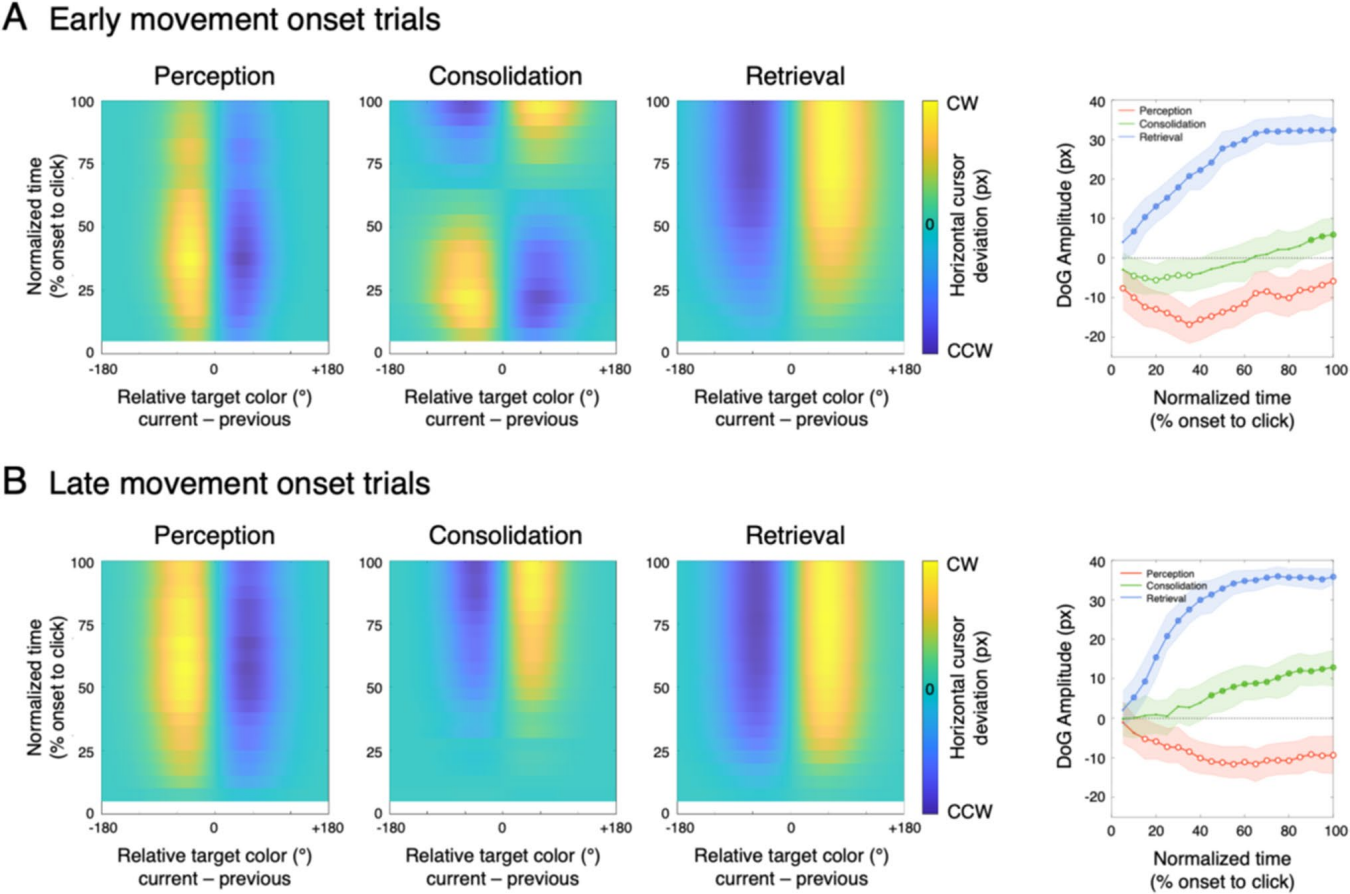
visualizes the estimated DoG fit curves as a function of normalized movement time for early and late movement onset trials, and the summary of the *α* parameter posteriors along with their HDI_95%_. Notably, early movement-onset trials during WM consolidation exhibited a distinct repulsion-to-attraction transition, with initial repulsive biases gradually shifting towards attraction as the movement progressed. In contrast, late movement-onset trials only showed the emergence of attractive bias, suggesting a more pronounced reliance on the mnemonic representation.

To further examine the unique pattern observed in consolidation reports, which may reflect a gradual transition from perceptual to WM-based processing, I analyzed the moment-by-moment horizontal deviation profiles along the progression of cursor movement. This was done by fitting to an extended hierarchical Bayesian DoG model that incorporates the time dimension (see *Data analysis* for detail). Additionally, I performed parameter estimation procedures separately for median-split datasets based on movement-onset latency in each report and participant.^3^ This approach is motivated by the assumption that early movement-onset trials in consolidation reports may rely, on average, more on perceptual representation while late-onset trials are more likely influenced by WM representation as consolidate progresses Fig. 4.

Together, these findings highlight the temporal evolution of serial biases, demonstrating that the transition from repulsion to attraction is not instantaneous but unfolds over time as perceptual representation consolidates into WM. This supports the hypothesis that serial biases are modulated by both sensory adaptation and mnemonic integration.

## Discussion

The present study investigated the temporal and process-specific dynamics of serial biases in visual perception and WM. Using a novel experimental paradigm that separated immediate perceptual reports from delayed WM consolidation and retrieval reports, I examined how serial dependence evolves across distinct processing stages. In particular, WM consolidation reports offered a detailed window to look into the gradual transition between perceptual and mnemonic processes, as reflected in changes in serial bias. Hierarchical Bayesian DoG modeling of continuous reports and mouse trajectories revealed three key findings: (1) a transition from repulsive bias in perceptual reports to attractive biases in WM reports; (2) within-trial trajectory dynamics in WM consolidation reports reflecting shifting shifts in representational reliance; and (3) stable individual differences in serial biases across different reporting types.

First, repulsion in immediate perceptual reports replicated prior findings under minimal WM demands (Fornaciai & Park, 2019). The continuous visibility of the target likely limited WM engagement, thereby isolating sensory-level mechanisms. This supports the idea that repulsive biases stem from early visual mechanisms that enhance sensitivity to change (Clifford et al., 2000; Fischer & Whitney, 2014). In contrast, attractive biases in consolidation and retrieval reports emerged as the influence of WM increased, with bias magnitude growing from consolidation to retrieval. These findings are consistent with the view that attractive serial dependence reflects a mnemonic process that integrates past and present to maintain perceptual stability (Kiyonaga et al., 2017; Liberman et al., 2014; Pascucci et al., 2019).

Second, mouse trajectory analyses provided fine-grained temporal resolution into the repulsion-to-attraction transition. In WM consolidation reports, initial movements exhibited repulsive biases that gradually shifted to attraction as the movement progressed. This effect was especially pronounced in trials with early movement onset, suggesting that participants initially relied on fleeting perceptual residues before WM integration took over. This temporal evolusion extends prior work by demonstrating that serial dependence unfolds dynamically over time as different cognitive processes come into play. These results also highlight the utility of movement-tracking methods for revealing the temporal evolution of cognitive processes (Park & Zhang, 2024b; Park et al., 2023; Scherbaum et al., 2010; Song & Nakayama, 2009).

Third, individual differences in DoG amplitude parameters revealed consistent cross-talk across the three report types. Individuals who exhibit stronger serial biases in one context tend to show similar tendencies in others. Such individual variability may reflect cognitive strategy differences or varying susceptibility to adaptation (Fischer & Whitney, 2014), with potential implications for tasks requiring precise perceptual or memory judgments.

Together, these findings support a dual-phase account in which repulsion and attraction reflect distinct but complementary mechanisms. The early repulsive phase appears to be driven by sensory adaptation while the later attractive phase likely reflects mnemonic integration (Moon & Kwon, 2022; Pascucci et al., 2019). This framework contrasts with accounts that attribute serial dependence solely to early sensory processes, instead suggesting that multiple cognitive systems, operating at different timescales, jointly shape the dynamic shift from repulsion during perception to attraction during WM. An alternative explanation, however, is that serial dependence arises from a single decision-making process in which prior-trial information is weighted adaptively based on current uncertainty (Chunharas et al., 2022). While this account may explain variability in the direction and magnitude of bias, follow-up examination (see Online Supplementary Materials) found no systematic relationship between behavioral uncertainty metrics (e.g., report precision, movement onset) and the direction of serial bias.

This study also extends serial dependence research to color feature, which has been rarely studied compared to orientation or spatial location (Barbosa & Compte, 2020). The results indicate that serial dependence in color follows similar patterns, supporting its domain-generality. While the well-documented influence of categorical bias remains a consideration for future work (Bae et al., 2014; Hardman et al., 2017), supplementary analyses revealed no evidence that categorical color boundaries introduced systematic distortions in continuous estimates or modulated the observed serial bias across color categories (see Fig. S2, Online Supplementary Material). That said, it remains an open question whether the observed repulsion-to-attraction transition generalizes across other feature domains. For example, Zhang and Luo (2023) reported that serial bias can manifest in a feature-dissociated manner. In their auditory categorization experiments, sensorimotor features such as pitch and motor responses were repulsed by past information, whereas abstract features like category choice were attracted toward past information. This finding implies that the past-present interaction may depend on feature type and task demands, a direction for future studies by extending the analytic approach to complex visual stimuli such as ensemble representations (Manassi et al., 2017).

Further neurophysiological investigations could clarify by which mechanisms the repulsion-to-attraction transition operates. For example, Fischer and colleagues (2024) observed attractive biases in multivariate neural patterns emerge at later stages of WM representations. Luo and colleagues (2024) demonstrated a two-stage “repulsive‐then‐attractive” sequence in neural activations. Specifically, the initial reactivation of past-trial information produces a repulsive effect on current sensory encoding that is subsequently followed by an attractive shift during decision-making. These neural findings dovetail with the behavioral dynamics observed here. However, whether serial biases reflect genuine representational shifts or post-perceptual decisional or read-out processes remains an open question (Sheehan & Serences, 2024; Zhang & Lewis-Peacock, 2024). Although this study did not dissociate stimulus and response contributions directly (Moon & Kwon, 2022; Sadil et al., 2024), follow-up analyses indicated that serial biases were more robust when aligned to the previous stimulus than to the previous response (Fig. S3, Online Supplementary Material). While prior studies have shown serial dependence even in the absence of overt responses (Fischer & Whitney, 2014; Fornaciai & Park, 2019), the lack of a response does not preclude a latent perceptual decision (Sadil et al., 2024). Thus, further work is needed to determine the contribution of unexecuted decision processes.

In conclusion, this study provides compelling evidence for the dynamic evolution of serial biases across perceptual and WM processes. By demonstrating a gradual transition from repulsive to attractive biases across behavioral responses at different cognitive processes, and a fine-grained temporal evolution of biases within individual trials, the findings support the view that attractive serial dependence originates from a mnemonic process that is jointly shaped by independent components of perceptual and mnemonic processes operating at different timescales. These findings have important implications for understanding how the brain maintains stable internal representations in the face of constantly changing input, with mouse trajectories offering a powerful tool to visualize cognitive dynamics in action.

## Supplementary Information

Below is the link to the electronic supplementary material.

Supplementary file1 (DOCX 3493 KB)

## Data Availability

Datasets generated and analyzed during the current study is available via the Open Science Framework repository at: https://osf.io/xem4w/?view_only=c16400ec980949989c5d0d500ece858f.

## Footnotes

1 While serial dependence has been extensively studied using visual-spatial features like orientation and spatial location, it has rarely been examined for a non-spatial color feature (Barbosa & Compte, 2020). This may potentially be due to its susceptibility to other types of biases such as categorical bias (Bae et al., 2014; Hardman et al., 2017; see Bae, 2024 for the role of cardinal bias in serial biases in orientation) and the fact that color is processed later in the visual hierarchy, potentially affecting sensitivity and reliability for multivariate analyses of neural data (Seymour et al., 2009; but see Hajonides et al., 2021).

2 In analyzing mouse-tracking data, various analytic choices must be made regarding how trajectories are aligned and compared across trials. For instance, raw trajectory data can be normalized by elapsed time or by distance to account for differences in movement duration or length (Park & Zhang, 2022). These methods are often chosen based on the specific research question and task paradigms, and while they may result in slightly different patterns in certain contexts, they often converge on similar conclusions. Additional analyses examining the potential influence of categorical color boundaries are reported in the Online Supplementary Materials.

3 The early versus late movement onset latencies were for perceptual (early 24% vs. late 45%), consolidation (early 22% vs. late 42%), and retrieval reports (early 33% vs. late 52%), *t*s > 12.4, *p*s <.001, Cohen’s *d*s > 2.85. Here the % unit represents the proportion of time at which movement onset occurred from the test cue onset to final clicking response.

## Code availability

The code utilized for data analysis and modeling in this study is available via the Open Science Framework repository at: https://osf.io/xem4w/?view_only=c16400ec980949989c5d0d500ece858f.
