# Supplementary material for: Process dynamics of serial biases in visual perception and working memory processes
Source: Psychon Bull Rev. 2025 May 27;32(6):2849–59. doi: 10.3758/s13423-025-02714-5 (PMC12627195; doi:10.3758/s13423-025-02714-5)
Supplement: Supplementary file 1 — Supplementary file1 (DOCX 3493 KB) [file 13423_2025_2714_MOESM1_ESM.docx]

**Supplementary Materials**

**S1. Comparison of Time-Based vs. Distance-Based Trajectory Normalization Methods**

The primary analyses normalized mouse trajectories based on time, to capture the temporal evolution of serial bias throughout different interval processes. An alternative approach is to normalize by spatial distance, such as from starting point to the final clicking response. Both methods have valid rationale depending on the specific hypothesis, and differences in outcome may or may not be critical (Park & Zhang, 2022).

To evaluate the results were sensitive to this choice, I utilized both normalization methods and compared the resulting DoG amplitude parameter estimates derived from area under the curve measures. As shown in Figure S1A, the two methods differ slightly in their sampling progressions. It may also be noteworthy that participants in the current dataset spent more than 65% of the total movement time before reaching the color-wheel, suggesting that initial motion phases were relatively slow and thus captures more of decisional dynamics. More importantly, the resulting posterior distributions of DoG amplitude estimates were highly similar across methods for all report types (Figure S1B). This confirms that the primary findings are robust to the choice of trajectory normalization method.

**Figure S1. Comparison of time-based and distance-based normalization methods for mouse trajectory analysis.** (A) Mean progression of normalized distance over normalized time across trials. The nonlinear shape indicates that participants spent the majority of time before reaching the color-wheel area. (B) Posterior distributions of DoG amplitude estimates for perceptual (red), consolidation (green), and retrieval (blue) reports, plotted separately for time-based (top) and distance-based (bottom) normalization. The similarity across methods confirms the robustness of serial bias estimates.

**S2. Control Analysis for Categorical Bias in Color Space**

While the main findings of the current study demonstrate a robust transition from repulsion to attraction in serial biases across processing stages, a non-spatial color features pose an additional concern due to its susceptibility to categorical biases. Prior research has shown that continuous color reports can exhibit systematic distortions near categorical boundaries (Bae et al., 2014; Hardman et al., 2017). If present, such distortions could confound the estimation of serial bias by introducing nonlinear biases that are unrelated to prior-trial effects.

***S2.1. Visual Inspection of Raw Report Distributions***

As an initial check, reported colors were plotted against their corresponding target colors across all trials, separately for the consolidation and retrieval reports (Figure S2A). These scatterplots did not reveal pronounced step-like clustering patterns that would be indicative of categorical biases (e.g., pulled toward categorical color center), but instead continuous. Minor symmetric clustering was observed but not in a manner that would produce systematic biases in one direction over the other.

***S2.2. DoG Modeling with a Model Layer of Color Category***

To further assess whether the observed serial bias effects were modulated by categorical boundaries, a follow-up analysis was conducted in which trials were grouped by the color category that the current trial’s target belonged. The circular color space was divided into 5, 6, or 7 equally spaced color bins, following conventions used in previous work (e.g., Bae et al., 2014). For each color bin, the serial bias magnitude was re-estimated using hierarchical Bayesian modeling of the DoG function. These analyses were performed on group-aggregated data due to the limited number of trials per color category and per relative distance condition.

**Figure S2. Control analysis for categorical bias in color space.** (A) Scatterplots of reported color vs. target color for all trials in the (left) consolidation and (right) retrieval conditions. (B) DoG amplitude estimates by color category for consolidation (left column, green) and retrieval (right column, red) reports, computed under 5-, 6-, and 7-category binning schemes (from top to bottom). Error bars represent 95% highest density intervals.

Figure S2B summarizes the resulting DoG amplitude estimates (means and 95% HDIs) for each color category across bin sizes. No individual category exhibited a qualitatively different pattern, nor was there evidence of systematic shifts or inversions of the serial bias direction across bins. These results suggest that the main serial dependence effects reported in this study are not substantially modulated by categorical boundaries in color space.

**S3. Serial Dependence Anchored by Previous Stimulus vs. Previous Response**

Recent studies suggest that serial dependence may be both anchored to previous responses as well as stimulus (Moon & Kwon; 2022; Sadil et al., 2024). While the present study was not specifically designed to dissociate these sources, follow-up analyses tested whether serial biases in consolidation reports were better aligned to the previous trial’s stimulus or response in retrieval reports. Figure S3A shows the mean error profiles computed relative to the previous stimulus and previous response, overlaid with hierarchical Bayesian DoG fits. The two curves appear largely similar, likely due to the relatively high accuracy of retrieval reports (circular SD = 23.8°). To further probe this question, the same analysis was repeated on a subset of trials in which the previous retrieval report deviated by more than ±20° from the target (approximately 24% of trials). As shown in Figure S3B, the serial bias pattern substantially diminished when aligned to the previous response, whereas the stimulus-anchored bias curve retained its systematic shape despite the smaller dataset. These findings suggest that, under the current task conditions, serial biases are more strongly anchored to the previous stimulus than to the previous response.

**Figure S3. Serial dependence computed relative to previous stimulus vs. previous response.** (A) Mean error as a function of relative color difference, computed with respect to the previous stimulus (green circles) and the previous response (red triangles), overlaid with hierarchical Bayesian DoG fits. (B) Same analysis restricted to trials where the previous retrieval report error greater than ±20° from the true target.

**S4. Alternative Single-Process Decision-Making Account**

An alternative interpretation of the present findings is that both repulsion and attraction may reflect a single decision-making process, in which observers dynamically adjust their reliance on previous trial information according to current uncertainty (Chunharas et al., 2022). According to this view, low uncertainty (e.g., visible perceptual targets) would prompt observers to emphasize differences between current and previous stimuli, resulting in repulsive bias. Conversely, higher uncertainty as in WM reports would lead observers to integrate previous trial information, producing attractive bias.

To evaluate this, I examined whether individual differences in perceptual bias direction were systematically associated with behavioral uncertainty. Specifically, I compared the five participants who showed attractive biases in the perceptual task with the five participants who showed the strongest repulsion. Neither reaction time nor movement onset time distinguished the two groups, and if anything, the attraction group initiated their movements slightly faster (normalized onset time: 28.2% ± 7.5% CI_95%_) than the repulsion group (30.6% ± 11.0% CI_95%_). Report precision measured by circular *SD* was also comparable across groups (attraction group: 10.3° ± 3.1°; repulsion group: 11.6° ± 2.2°; full sample: 10.6° ± 1.4°). These results do not support the idea that perceptual uncertainty drives attraction.

Moreover, strategic avoidance of prior-trial colors is unlikely given the randomized color-wheel orientation and varied inter-trial color differences. Prior work using similar paradigms has shown attractive serial biases even when the target was fully visible and the prior color was predictable (Park & Zhang, 2024), challenging the assumption that low uncertainty leads to repulsion. Taken together, while adaptive uncertainty-based weighting may play a crucial role in some contexts such as between-item interactions among concurrent memoranda, the current results are more consistent with a dual-process account in which early sensory adaptation drives repulsion and later mnemonic integration supports attraction.

**References**

Bae, G. Y., Olkkonen, M., Allred, S. R., Wilson, C., & Flombaum, J. I. (2014). Stimulus-specific variability in color working memory with delayed estimation. *Journal of Vision, 14*(4), 7.

Chunharas, C., Rademaker, R. L., Brady, T. F., & Serences, J. T. (2022). An adaptive perspective on visual working memory distortions. *Journal of Experimental Psychology. General, 151*(10), 2300-2323.

Hardman, K. O., Vergauwe, E., & Ricker, T. J. (2016). Categorical working memory representations are used in delayed estimation of continuous colors. *Journal of Experimental Psychology. Human Perception and Performance, 43*(1), 30-54.

Moon, J., & Kwon, O. S. (2022). Attractive and repulsive effects of sensory history concurrently shape visual perception. *BMC biology, 20*(1), 247.

Park, H., & Zhang, W. (2024). The dynamics of attentional guidance by working memory contents. *Cognition, 242*, 105638.

Park, H., & Zhang, W. (2022). Trial-by-trial mouse trajectory predicts variance in precision across working memory representations: A critical reanalysis of Hao et al. (2021). *Psychonomic Bulletin & Review, 29*(6), 2181-2191.

Sadil, P., Cowell, R. A., & Huber, D. E. (2024). The push–pull of serial dependence effects: Attraction to the prior response and repulsion from the prior stimulus. *Psychonomic Bulletin & Review, 31*(1), 259-273.
